# Valedictory Address in Behalf of the Graduating Class of 1860

**Published:** 1860-10

**Authors:** S. T. Beasly

**Affiliations:** LaGrange, Ga.


ATLANTA

Medical and Surgical Journal.

Vol. VI.] OCTOBER, 1860. [No. 2.

### ORIGINAL COMMUNICATIONS.

### ARTICLE I.

*Valedictory Address in behalf of the Graduating Class of* 1860. By S. T. Beasly, of LaGrange, Ga.

*Respected Faculty of the Atlanta Medical College:*

To me has been assigned by my class-mates the duty of bidding you farewell. And be assured it is with the most profound self-distrust that I undertake this important mission. For what can I bring worthy of your acceptance, and of theirs? Yet, however feeble my voice, I feel it is but a duty to obey the call of my fellow-classmates; and this sentiment which now glows within my bosom will, I trust, be an incense to the offering, and consecrate it to your acceptance. The many ties which have bound us together as teacher and student, during our short but pleasant association in this institution are about to be dissolved. From the safe and protecting enclosure of your college—from your kind and fatherly counsels, we have now to go forth into life. Into life checkered with trials and troubles, with pleasures and enjoyments—while your course is—

"Still onward through the gloom,
The sunshine or the shade, thy task is still
To guide more feeble spirits to the fount
Of learning and of truth, from whence thine own
Hath drawn large floods of light and wisdom forth."

The Atlanta Medical College is still in its infancy. Yet, under your fostering care, it is enabled at this time to compete successfully with any like institution within the limits of this broad Union. What higher honor can there be than "to teach the young idea how to shoot"—to uncover the gems of beautiful thought that lie buried in obscurity? You, respected faculty, have faithfully endeavored to give us the knowledge to unravel the mysteries of our every-day occupation, and to adorn the station in life to which we have been called. You have endeavored to give us the foundation upon which the fabric is to be raised. If, then, we henceforth meet, with fortitude and confidence, the vicissitudes of fortune, it is to your noble and untiring ardor, your kind and parental indulgence, your self-sacrificing disinterestedness, that we will owe it all. The parting word sounds sadly upon our ear, yet, we must say "farewell." And wherever, in this wide world, our lots may be cast, we will ever turn with *grateful remembrance* to you; and when death flings his sable mantle over the drama of your lives, your memories will ever linger upon the hearts of those you have left behind; and the dew-drops falling from the altar of gratitude, will ever keep fresh the green turf that shall cover you—once more, "farewell."

To you, respected auditory, I now turn in behalf of the graduating class of "eighteen sixty." We have before us to-day the patrons of learning and of truth. The fond father whose sands of life having well nigh run out, and who now only ambitious for a hopeful son, is here. Mothers, brothers, and sisters, are here, with their warm smiles to greet him who returns to their embrace with his blushing honors thick upon him ; and last, though not least, we have her, whose gentlest smile is the fairy's wand that causes instant submission—her to whom even the physician will always come, with fevered brow, to ask the medicine of love to cure "*his* ills." All, all, are here for the purpose of welcoming into your ranks those who promise to do good in the service of their country and their God. It is a time when *hope* beats with as strong a pulse in the hearts of the old as the young, and, therefore, fitting that we should present to you our most heartfelt thanks for the encouraging smiles with which you have greeted us on this occasion. And we leave you with a grateful appreciation of the many kind and friendly hospitalities which you, as the citizens of Atlanta, have always extended to us ; and though our future paths may be far apart, yet sweet memory, wafted by the gentle gale, will oft revisit and linger with increased delight upon the scenes we to-day leave behind us. Farewell.

---

What can I say to *you*, fellow-classmates, that shall be worthy the theme and the occasion ? As I look around and behold your familiar faces radiant with smiles of happiness, a spirit of sadness steals upon me; I know that we must part, some, perhaps, never to meet again. Would, then, that I could roam the fields of literature and of scince, that I might gather a boquet fragrant enough to be laid upon this sacred shrine. We have arrived at a stage in the journey of life, from which another advancing step will place us in the world's great theatre, and we are all looking forward with joy and hope to the scenes that there await us.

"Bright eyes are beaming on us now,
Fond lips are wreathing many a smile;
The light of hope is on each brow,
And whispering to our hearts the while."

Yet, fellow-classmates, in the profession we have chosen, we may expect to meet with thorns and poisons, as well as fruits and flowers. Barren deserts, vast marshes and rocky mountains will abound, as well as fertile plains and blooming valleys. We will meet with men whose every thought and action are guided alone by self-interest and self-promotion—men who will attempt, by unworthy means, to destroy the character or ruin the reputation of those whom they can not equal in the race for honor or distinction. We will find the doctrine of "Quacks," delusions made up by the ravings of the maniac, the folly of the fool or the treachery of the knave, seeking to undermine the pillars of our holy temple. But let us not darken the picture by our gloomy shadows. Let us leave that task to time, who knows too well his duties and his occasions. Our's is indeed a noble country:—its bold streams, broad pararies and lofty mountains are enough to elicit alike our poetry and our patriotism. Here the Hydra-headed monsters, ignorance and superstition, may, for awhile, obscure the light of science, but sooner or later it is destined to burst forth with increased brilliancy and beauty. Let us, then, fellow-classmates, tread on in the old beaten path of virtue and of truth. Let us keep midway between the sluggishness of ignorance and the ill-timed energy of vice. Let us,

"Catch then, Oh! catch the transient hour,
Improve each moment as it flies;
Life's a short summer—man a flower—
He dies, alas! how soon he dies!"

and lastly, let us ever cherish a likely remembrance of the paternal solicitude and enlightened councels which we have here had the privilege of enjoying; and even amid the hurry and bustle of life, when we are draining the guilded cup with which pleasure allures her votaries, or struggling with the calamities which befall the human race, may we ever turn with fond recollections to the "Atlanta Medical College." My task is done. I again return to you my most sincere thanks for the honor you have conferred upon me. May heaven shed its richest blessings upon your heads, and in journeying down the path of life, may you never tread under foot, the delicate little flower "Forget-me-not."

